# The impact of constitutive mTORC1 hyperactivity on structural synaptic plasticity and social behaviour under standard conditions and environmental enrichment

**DOI:** 10.1192/j.eurpsy.2022.506

**Published:** 2022-09-01

**Authors:** S. Granak, K. Tuckova, V. Kutna, I. Vojtechova, L. Bajkova, T. Petrasek, S. Ovsepian

**Affiliations:** National Institute of Mental Health, Department Of Experimental Neurobiology, Klecany, Czech Republic

**Keywords:** animal models of autism, dendritic spines, tuberous sclerosis complex, enriched environment

## Abstract

**Introduction:**

Autism spectrum disorders (ASD) are a group of neurodevelopmental disabilities causing major social, communication, and behavioural challenges. Although causative roles of altered genes and environment are recognized, the underlying mechanisms remain elusive.

**Objectives:**

We carried out a longitudinal analysis of morphological correlates of synaptic plasticity in the cortex with an array of neuro-behavioural tests in *Tsc2* loss-of-function ASD rats with persistent mTORC1 hyperactivity.

**Methods:**

Dendritic spine density and morphology with astroglial response were analysed along with behavioural tests in 45 d.o., 90 d.o. and 12 m.o. age groups maintained under standard or enriched conditions.

**Results:**

We report a higher density of spines, with a bigger proportion of thin spines in 90 d. o. *Tsc2+/-* rats, while under enrichment the spine density in 12 m.o groups was lower. In behavioural tests, rats under enrichment showed higher activity in open arms and anogenital contact tests in the second and third age groups. They also showed enhanced self-grooming. Total distance travelled in the open field by *Tsc2+/-* rats was less in the first and second age groups. Confocal imaging showed an increase in pS6 expression in second and third *Tsc2+/-* groups, implying mTORC1 hyperactivity.

**Conclusions:**

Our results show that the environment may have differential neuro-behavioural impacts in rats with unleashed mTORC1, in agreement with the two-hit mechanisms of the underlying neuro-behavioural effects in this model. Although the results of the morphometric analysis suggest a causal link between behavioural changes with altered synaptic plasticity, the mechanisms and involved signalling remain to be defined.

**Disclosure:**

No significant relationships.

